# DrugRepo: a novel approach to repurposing drugs based on chemical and genomic features

**DOI:** 10.1038/s41598-022-24980-2

**Published:** 2022-12-07

**Authors:** Yinyin Wang, Jehad Aldahdooh, Yingying Hu, Hongbin Yang, Markus Vähä-Koskela, Jing Tang, Ziaurrehman Tanoli

**Affiliations:** 1 Research Program in Systems Oncology, Faculty of Medicine, University of Helsinki, Helsinki, Finland; 2 Department of Chemistry, University of Cambridge, Cambridge, UK; 3 Institute for Molecular Medicine Finland, University of Helsinki, Helsinki, Finland; 4 BioICAWtech, Helsinki, Finland

**Keywords:** Computational biology and bioinformatics, Molecular medicine

## Abstract

The drug development process consumes 9–12 years and approximately one billion US dollars in costs. Due to the high finances and time costs required by the traditional drug discovery paradigm, repurposing old drugs to treat cancer and rare diseases is becoming popular. Computational approaches are mainly data-driven and involve a systematic analysis of different data types leading to the formulation of repurposing hypotheses. This study presents a novel scoring algorithm based on chemical and genomic data to repurpose drugs for 669 diseases from 22 groups, including various cancers, musculoskeletal, infections, cardiovascular, and skin diseases. The data types used to design the scoring algorithm are chemical structures, drug-target interactions (DTI), pathways, and disease-gene associations. The repurposed scoring algorithm is strengthened by integrating the most comprehensive manually curated datasets for each data type. At DrugRepo score ≥ 0.4, we repurposed 516 approved drugs across 545 diseases. Moreover, hundreds of novel predicted compounds can be matched with ongoing studies at clinical trials. Our analysis is supported by a web tool available at: http://drugrepo.org/.

## Introduction

The average cost of developing a new drug is billions of dollars, and it takes about 9–12 years to bring a new drug to the market^1^. Finding new uses for approved drugs has become a primary alternative strategy for the pharmaceutical industry. This practice usually referred to as drug repositioning or drug repurposing, is highly attractive because of its potential to speed up the process of drug development, reduce costs, and provide treatments for unmet medical needs^2^. In this regard, compounds that have passed through phases I or II in the drug discovery pipeline but never make it to the market due to efficacy issues carry great potential for drug repositioning. Traditionally, drug repurposing success stories have mainly resulted from opportunistic and serendipitous findings^3^. For example, sildenafil citrate was originally developed as an antihypertensive drug but later repurposed by Pfizer and marketed as Viagra to treat erectile dysfunction based on retrospective clinical experience, leading to massive worldwide sales. Other examples of such drug repositioning include cancer drugs: crizotinib, sorafenib, azacitidine and decitabine, all of which failed to reach the markets in their initial indications yet now are essential tools in the treatment of various types of cancers^4^. Over recent years, various computational resources are developed to support systematic drug repurposing. Popular information sources for *in-silico* drug repurposing include, for instance, electronic health records, genome-wide association analyses or gene expression response profiles, pathway mappings, compound structures, target binding assays and other phenotypic profiling data^3^. Several systematic review articles on the use of computational repurposing approaches are available that cover machine learning (ML) algorithms^5–7^. Several databases directly support *in-silico* drug repurposing, including Drug Repurposing Hub^8^, repoDB^9^ and RepurposeDB^10^. On the other hand, hundreds of databases can indirectly support drug repurposing^7,11^. However, these databases provide experimentally tested indications only for a limited number of approved drugs and ignore the massive number of investigational compounds that could be potential candidates for drug rescuing. Drug-target profiles for approximately two million such preclinical and investigational compounds are available at ChEMBL^12^ and other databases.

Drug-target interactions (DTI), meaning the target molecules each compound binds to and the relative binding strength and impact on cellular functions, lie at the heart of drug discovery and repositioning. Several artificial intelligence (AI) methods for drug repurposing are based on DTIs as well as chemical structural similarities^13–15^. Computational approaches are primarily data-driven and involve a systematic analysis of several components (or data types) before suggesting a repurposed indication. These components may include chemical structures, adverse event profiles, compound-target interactions, pathways, disease-gene associations, genomics, proteomics, and transcriptomics information. Drug repurposing methods can be developed based on the individual or combination of these components. PREDICT is currently the second most cited computational method for predicting drug-disease relationships^16^. It is based on drug-drug and disease-disease networks based on 593 drugs and 313 diseases. Li, Jiao et al. proposed a bipartite graph-based method to identify new drug indications through its relationship with similar drugs^17^. The CMAF method uses matrix factorization to predict drug-disease associations based on drug and disease similarity networks^18^. However, these methods are applied only to a limited set of drugs resulting in limited prediction outcomes. Also, there is no such interactive web application for these methods.

Therefore, in this study, we propose DrugRepo (http://drugrepo.org/); a novel scoring algorithm that can effectively repurpose approved drugs as well as rescue hundreds of thousands of investigational compounds based on three components, including (1) overlapping compound-target score (OCTS), (2) structure similarity score based on Tanimoto coefficient (TC), and (3) compound-disease score (CDS). The first component (OCTS) is the Jaccard index between drug-target profiles of approved drugs and candidate investigational compounds, as explained in section "Compound-target profiles". The second component is based on structural similarity between fingerprints of the approved drugs and candidate compounds using the Tanimoto coefficient. The third component is based on disease-gene associations and protein–protein interactions data. Finally, the DrugRepo score is the average of the three component scores. We have collected approved indications 1091 drugs, and 669 diseases from clinical trials (https://clinicaltrials.gov/). The Potent compound-target profiles are collected for 0.8 M compounds. To explore the translational impact of DrugRepo, we have cross-referenced candidate compounds with completed clinical trials. We have observed that 186 compounds are explored in different clinical studies across nine cancer types. We have compared our candidate compounds with the predicted compound disease relationships in Comparative Toxicogenomic Database (CTD)^19^ and found a statistically significant overlap.

We have showed through several examples that the DrugRepo score ≥ 0.4 is an appropriate threshold to predict new drug-disease relationships. Using DrugRepo score ≥ 0.4, we have found repurposing potential for 516 approved drugs across 545 diseases. We have demonstrated the use case of DrugRepo’s GUI to repurpose drugs for chronic myeloid leukaemia. As per our knowledge, no such tool can compute drug-disease relationships on a wide scale. These promising findings demonstrate the versatility of DrugRepo and provide a quick and effective scoring method for drug repurposing.

## Materials

Several data types are integrated into this analysis, e.g., approved drug indications, compound-target profiles, disease-gene associations, and protein–protein interaction (PPI) networks. These data types are consequently explained in the following subsections.

### Approved drug indications

Approved drug indications are extracted from the clinical trials database, as it is the most up-to-date repository for drug indications and clinical phases for the compounds. However, the data provided by clinical trials is not well structured and doesn’t provide standard naming conventions or identifiers for the compounds and diseases. We, therefore, utilized a semi-automated approach to extract drug-disease indications and assigned UML-CUI and standard InChIKey identifiers for drugs and diseases, respectively. The standard InChIKey mapping is performed using the PubChem python client, whereas UML-CUIs are assigned to the diseases using disease annotations provided by DisGeNET^20^. The curated gold standard dataset contains 669 diseases (from 22 groups), 1091 drugs, and 3757 approved drug indications, as shown in Supplementary file 1.

### Compound-target profiles

Non-overlapping compound-target profiles are extracted from the five most comprehensive (and manually curated) databases, namely, ChEMBL^21^, BindingDB^22^, GtopDB^23^, DrugBank^24^, and DGiDB^25^. The compound identifiers in these databases are mapped into standard InChIKeys and SMILES using UniChem^26^ and PubChem^27^, respectively, whereas identifiers for target proteins are mapped to UniProt identifiers^28^. The combined non-overlapping compounds from the five databases exceed 2M with approximately 15,000 targets. These comprehensive datasets are extracted using the application programmable interfaces (APIs), standalone text files, and SQL dumps. The first three databases, ChEMBL, BindingDB and GtopDB, provide quantitative bioactivity data, such as measurements in terms of IC_50_, K_d_, and K_i_, whereas DrugBank and DGiDB contain unary but experimentally verified compound-target interactions. In addition to active or potent compound-target profiles in ChEMBL, BindingDB and GtopDB, there exists a big proportion of in-active compound-target profiles (concentration > 10,000 nM). These in-active compound-target profiles could jeopardize the analysis in the proposed research. Therefore, in this analysis, we consider only potent compound-target profiles (concentration is ≤ 1000 nM)^29^. Hence, we have left with 788,078 compounds and 8754 protein targets. Potent target profiles for these ~ 0.8 M compounds are already integrated and publicly available in MICHA^30^.

### Disease-gene associations and PPI networks

To support the large-scale drug repurposing, we integrated manually curated disease-gene associations from DisGeNET^20^. There are 9,703 genes, 11,181 diseases and 84,038 associations. These curated disease-gene associations are provided in Supplementary file 2.

We have downloaded the protein–protein interactions (PPI) data from Cheng et al.^31^. They have manually curated high-quality human interactome based on five experimental types, (1) PPIs tested by high-throughput Y2H systems, (2) kinase-substrate interactions by literature-derived low-throughput experiments, (3) kinase-substrate interactions from high-throughput experiments, (4) curated PPIs identified by affinity purification and (5) signalling network by literature-derived low-throughput experiments. They have collected PPIs from databases such as IntAct^32^, InnateDB^33^, PINA^34^, HPRD^35^, MINT^36^ and BioGRID^37^. So, instead of collecting from a particular database, we preferred to use the precompiled PPIs in Cheng et al. The extracted dataset contains 16,677 proteins and 243,603 PPIs.

## Methods

**Figure 1:**
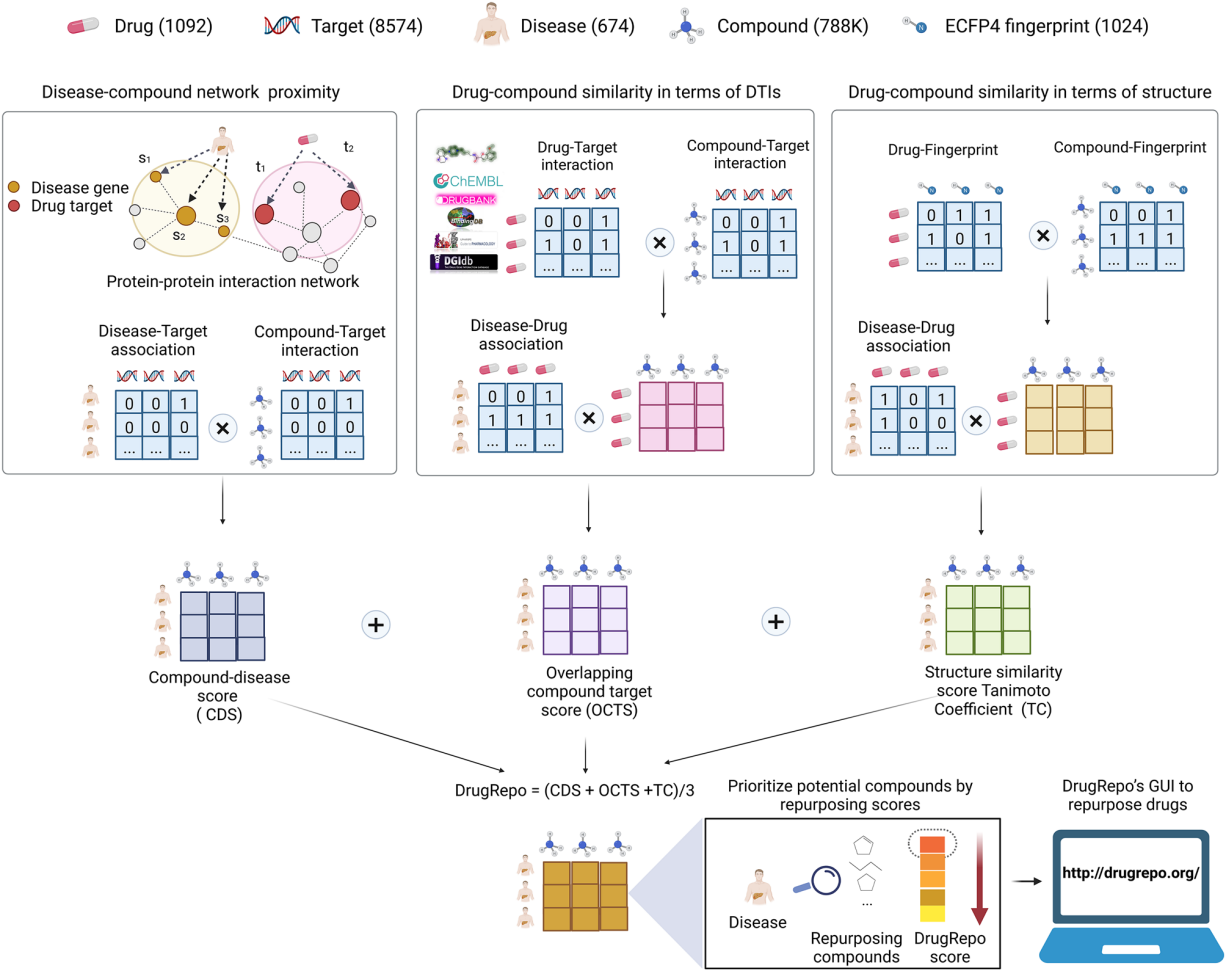
The schematic figure for drug repurposing in DrugRepo. The DrugRepo pipeline starts with the user selecting a particular disease. There are ~ 0.8 M candidate compounds in DrugRepo that can be repurposed or rescued for 669 diseases. At first, the pipeline finds approved drug(s) for the selected disease and searches for structurally similar drugs or compounds. In this step, the Tanimoto coefficient (TC) describes the structural similarity between molecular fingerprints (ECFP4) of approved and candidate compounds. A threshold is used to favor similar molecular structures. The second step is to compute DTI profiles for candidate compounds and approved drugs. The OCTS is the score based on overlapping DTIs between approved and candidate compounds. In case of multiple approved drugs for a disease, we took average of OCTS and TC scores. The third step is to compute the compound-disease score (CDS). The CDS is the average of the minimum distances in the PPI networks between target molecules and molecules associated with the selected disease. The average distance is normalized to 0–1. Finally, the DrugRepo score is calculated as the average of the three component scores. The higher the DrugRepo score between the approved drug and the candidate compound, the higher the possibility of repurposing the compound for a particular disease. Finally, we developed the DrugRepo’s GUI to provide a user-friendly service for repurposing drugs with our pipeline. The OCTS between approved and candidate compounds are computed using Eq. 1. The OCTS ranges from 0 to 1 and represents the proportion of targets shared between an approved drug and the candidate compounds. Candidate compounds sharing more targets with the approved drugs will have higher OCTS values.

There are 788,078 compounds for which at least one potent target (concentration is ≤ 1000 nM) exists in any of the five DTI databases. We call these agents candidate compounds to be rescued or repurposed. For each candidate compound, the DrugRepo score is computed as the average of three component scores, OCTS, TC, and CDS, derived from comparing each candidate compound to 1091 approved drugs (Fig. 1). Similarly, we have computed DrugRepo scores between approved drugs for a particular disease and drugs approved for other diseases (drug repurposing). We have considered only those cases where the structural similarity between the approved drug and candidate compound is ≥ 0.2. This way, we have been left with 2,207,367 scores.

The overlapping compound-target score **(**OCTS) is the Jaccard index between drug-target profiles of approved drugs and candidate compounds and is computed using Eq. 1.

1 $$ OCTS = \frac{{Drug_{T} { } \cap {\text{ Compound}}_{T} }}{{\min \left( {\left| {Drug_{T} } \right| ,\left| {{\text{Compound}}_{T} } \right|} \right)}} $$

where $Drug_{T} and {\text{Compound}}_{T}$ are the sets of potent targets for a pair of approved drugs and candidate compounds, respectively. Similarly,$\left| {Drug_{T} } \right| and \left| {{\text{Compound}}_{T} } \right|$ are the total number of targets associated with the approved drug and the candidate compound, respectively.

**Figure 2:**
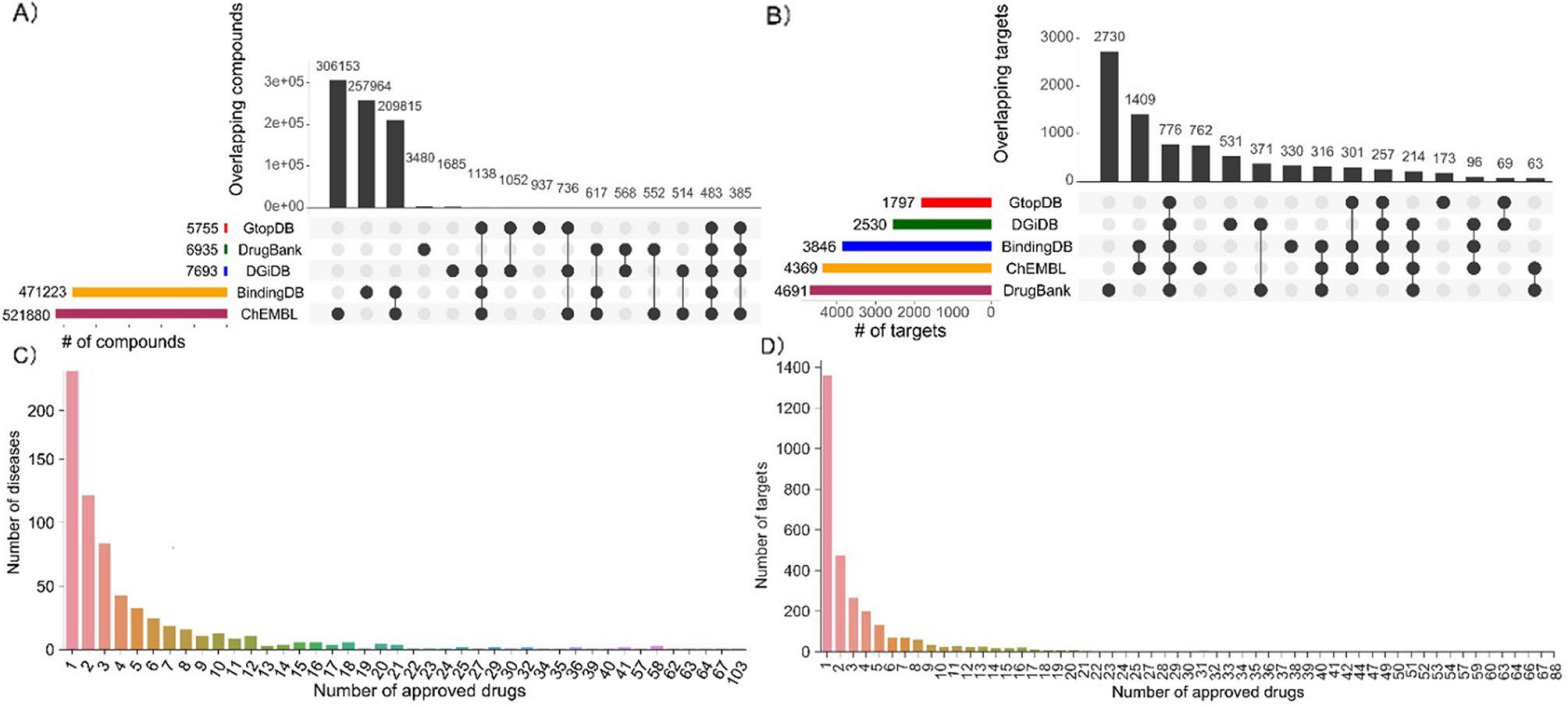
(**A**) Number of overlapping compounds across five databases. (**B**) Number of overlapping targets between five databases. (**C**) Distribution of diseases across 1091 approved drugs. (**D**)Target coverage across 1091 approved drugs.

Compound-target profiles are extracted from five databases. The number of overlapping compounds and targets in these five databases are shown in Fig. 2A, B, respectively. There are 483 compounds and 776 targets that are common in all five databases. As evident from Fig. 2A, ChEMBL features the most comprehensive collection of compounds, whereas DrugBank covers a more significant number of targets (Fig. 2B).

Drug repurposing is challenging because of shortcomings in data coverage. The diseases associated with a significant number of approved drugs may have better chances of correctly repurposing the compounds as the number of candidate compounds is larger. However, only very few diseases are associated with more approved drugs. HIV is associated with the highest number of approved drugs (n = 103), but as shown in Fig. 2C, more than 70% of diseases have less than five approved drugs. On the other hand, the lack of drug-target interactions is also a hurdle, as it limits the matching of compounds by the putative mechanism of action. Indeed, most approved drugs have less than 30 potent targets (Fig. 2D). To compensate for the shortage of approved drugs and drug-target-interactions, we incorporated two additional components in the DrugRepo pipeline: the Tanimoto coefficient (TC), which is a similarity score based on 2D structures, and the compound-disease score (CDS), which ranks new compounds based on how closely their target spaces match with the target proteins that are associated with the diseases.

The Tanimoto coefficient (TC) measures structural similarities between molecular ECFP4 fingerprints of approved and candidate compounds for a particular disease. The fingerprints are the bit strings denoting the presence or the absence of chemical substructures and are calculated using the RDKit package^38^.

2 $$ {\text{TC}} = \frac{{N_{AC} }}{{N_{A } + N_{C } - N_{AC} }} $$

where $$N_{A } and N_{C } $$ are the number of sub-structures present in the approved drug and candidate compound, respectively, and $$N_{AC}$$ are the number of common sub-structures found in both the approved drug and the candidate compound. The value of TC is between the range 0–1 and constitutes the second component of DrugRepo.

The compound-disease score (CDS) is measured by averaging the minimum distances in PPI networks between potent targets of the candidate compound and genes associated with the disease, as shown in Eq. 3. Curated disease-gene associations are extracted from DisGeNET for the 669 diseases used in this analysis. There are 22,399 non-zero CDS values.

3 $$ CDS\left( { C_{G} ,D_{G} } \right) = \frac{1}{{\left| {D_{G} } \right| + \left| {C_{G} } \right|}}\mathop \sum \limits_{i,j} min d\left( {g_{i} ,g_{j}^{\prime } } \right) $$

where $$C_{G} = \left( {g_{1, } g_{2, } ...} \right)$$ is the set of gene targets for candidate compounds and $$D_{G} = \left( {{\text{g}}_{1}^{^{\prime}} ,{\text{g}}_{2}^{^{\prime}} ...} \right)$$ are the genes associated with a particular disease (acquired from DisGeNET). The average of minimum distances between $$C_{G} and$$ $$D_{G} $$ are computed in PPI networks. The average distance is further normalized to 0–1 using min–max normalization. Finally, the DrugRepo score is the mean of the three compound scores and ranges from 0 to 1. The higher the DrugRepo score between an approved drug (for a particular disease) and a candidate compound, the greater the repurposing potential of the candidate compound for that disease.

## Results

To explore the translational impact of DrugRepo, we have evaluated our repurposed drugs or rescued candidate compounds using two methods, i.e., (1) cross-referencing of thousands of the rescued compounds using disease-compound associations in CTD^19^, (2) matching 186 compounds across nine cancer types for which either Phase I or Phase II trials have been completed or Phase III trials is ongoing.

### Matching repurposed compounds with disease-compound associations in CTD

The Comparative Toxicogenomic Database (CTD) contains manually curated and inferred compound-disease relationships^19^. CTD associates thousands of compounds with diseases based on drug-target and disease-gene relationships. Our scoring method and the datasets differ from CTD, but the output types in DrugRepo and CTD are the same. We, therefore, assessed the accuracy of DrugRepo by comparing the repurposed compounds with compound-disease relationships in CTD. We have downloaded disease-compound relationships from CTD. In CTD, compounds are represented by CAS identifiers or compound names; and diseases using Mesh ids. To make the comparison possible, we have mapped compound names from the CTD dataset into standard InChIKeys using PubChempy. Diseases are mapped from MESH ids into UML-CUI. There are 1,048,548 compound-disease associations in CTD, including 3941 compounds and 6119 diseases. Several of these compounds and diseases cannot map into standard InChIKeys and UML-CUI identifiers. We, therefore, skipped those cases and have left with only 168,471 compound-disease associations (605 diseases and 2,598 compounds), as shown in Supplementary file 3. These associations are used to match rescued compounds or repurposed drugs by DrugRepo. The significance of matching results for overlapping compounds (for a particular disease) between DrugRepo and CTD is computed using the following equations:

$$ \begin{aligned} N_{overlap} & = \left| { C_{d} \cap C_{r} } \right| \\ N_{expected} & = |C_{d} \left| * \right|C_{r} \left| {/|C_{all} } \right| \\ Sig_{d} & = N_{overlap} - { }N_{expected} \\ \end{aligned} $$

where $$C_{all}$$ is the set of all candidate compounds (~ 0.8 M) in DrugRepo, $$C_{d}$$ is the set of compounds associated with disease ‘d’ in CTD, $$C_{r}$$ is the set of repurposed compounds by DrugRepo for the same disease ‘d’$$, N_{overlap}$$ is the number of overlapping compounds between CTD and DrugRepo, $$N_{expected}$$ is the expected number of compounds overlapping with $$C_{d} $$ if we randomly choose the same number of compounds ($$|C_{r} |)$$ from $$C_{all} ,{\text{ and }}Sig_{d}$$ is the significance of matched compounds. If the significance is greater than 0, our repurposing is not random.

**Figure 3:**
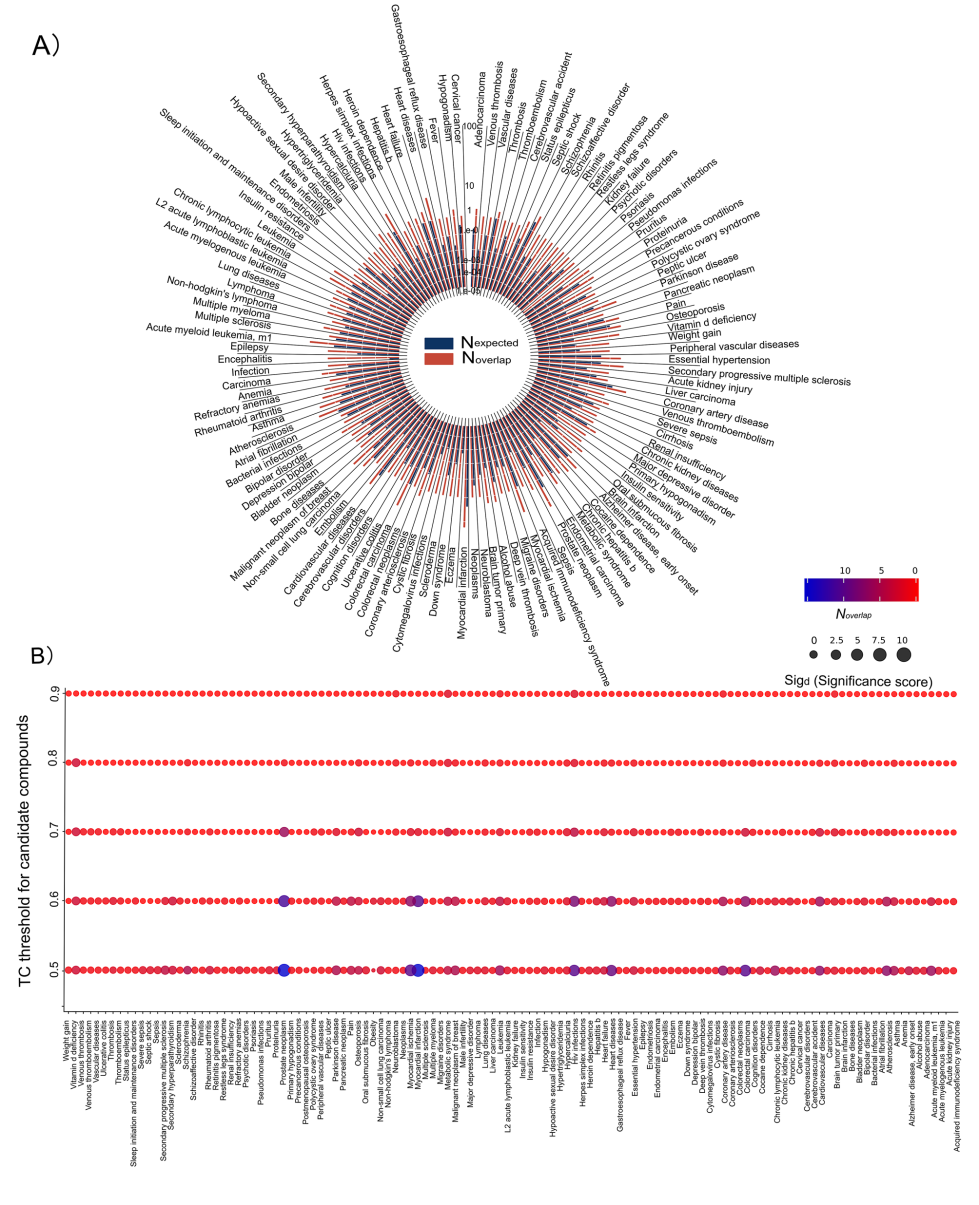
(**A**) The circular bar shows drug repurposing results matched with CTD. All the matched diseases were distributed as pie in the circle. The blue bars represent $({N}_{expected}$) the expected number of compounds, if chosen randomly while the red bar represents the actual overlap (N_overlap_) between compounds in CTD and DrugRepo. (**B**) The Significance scores at different TC thresholds (with Y-axis as thresholds and X-axis as a disease whose true positive is not 0). The significance score is represented by dot size, and the colour from red to blue represents number of overlapping compounds.

DrugRepo contains ~ 0.8 M candidate compounds and 1,091 approved drugs. This could result in a vast matrix (~ 800 M). Therefore, we have considered only those candidate compounds with at least 50% structural similarity with the approved drugs (TC ≥ 0.5). If $N_{overlap}$ for a particular disease is greater than $N_{expected}$, ${ }Sig_{d}$ is positive, meaning that repurposed or rescued compounds by DrugRepo are matched with CTD. Diseases with significance scores > 0 are shown in Fig. 3A ($N_{expected}$ is shown with blue and $N_{overlap}$ with red bars). As shown in Fig. 3A, the blue bar represents the expected number of compounds, and the red bar indicates the actual number of matched compounds. Most blue bars have significance scores less than 1, suggesting that if chosen randomly, there should be less than one candidate compound that can match most diseases. We have matched more than one correctly repurposed compound for 118 diseases (Fig. 3A). In other words, DrugRepo shows good repurposing and rescuing ability in 118 of the 605 diseases in CTD, suggesting that our novel pipeline effectively repurposes drugs for several diseases. For example, DrugRepo contains 4921 candidate compounds and CTD has 401 compounds for myocardial infarction. If the selection of the 4921 compounds (from the 788,078 total candidate compounds) has been random, we would have expected an average of 2.51 matched compounds. However, we have matched 15 compounds with CTD (6 folds bigger than the expected number), suggesting that the DrugRepo pipeline extracts biologically relevant candidate compounds. The details of overlapping drug-disease relationships and expected scores for 569 diseases are provided in Supplementary file 4.

To investigate the effect of structural similarity on drug repurposing/rescuing, we have evaluated the matched repurposed compounds on five different thresholds (TC: 0.5, 0.6, 0.7, 0.8, 0.9). As shown in Fig. 3B, the number of matched compounds tends to decrease with strict TC filtration on repurposed compounds, as expected. However, the significance scores are also reduced, especially after TC ≥ 0.9, suggesting that high structure similarity is not a determining factor for drug repurposing. Many of the matched repurposed compounds are located at TC ≥ 0.5. On the other hand, the number of matched compounds and significance scores is relatively stable between 0.6 ≤ TC ≤ 0.7. Therefore, TC values between 0.6 and 0.7 might be optimal for drug repurposing.

**Figure 4:**
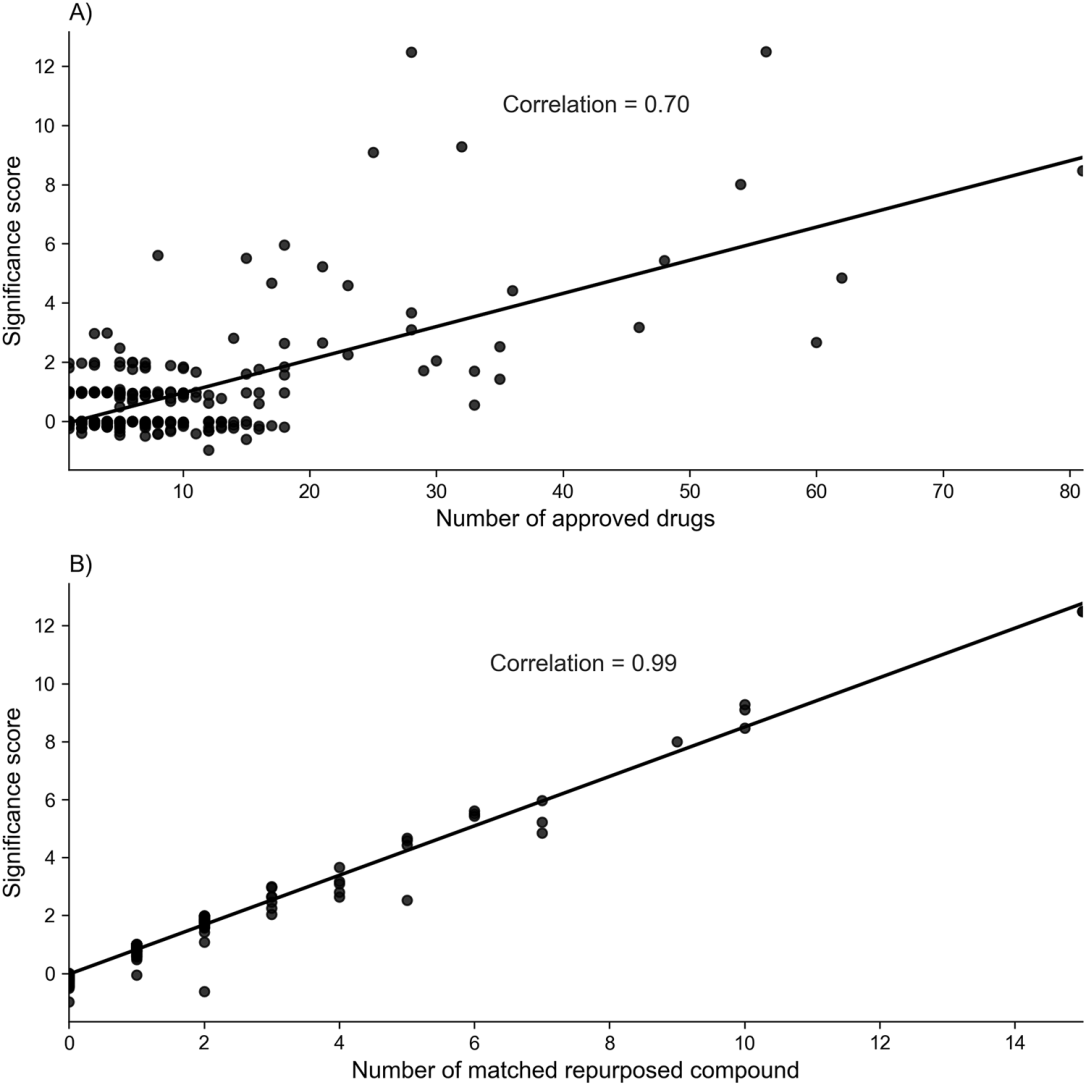
The impact of significance score on number of: (**A**) approved drugs associated with a disease, (**B**) matched repurposed drugs or recued compounds.

We also have analyzed whether diseases associated with a more significant number of approved drugs can affect the DrugRepo scoring. As shown in Fig. 4A, if a specific disease is associated with a considerable number of approved drugs, more repurposed compounds can be matched (Pearson correlation = 0.7). Similarly, the number of matched repurposed compounds (N_overlap_) is also closely associated with the significance score (Fig. 4B). Conversely, DrugRepo performance remains poor for the diseases associated with fewer approved drugs.

**Figure 5:**
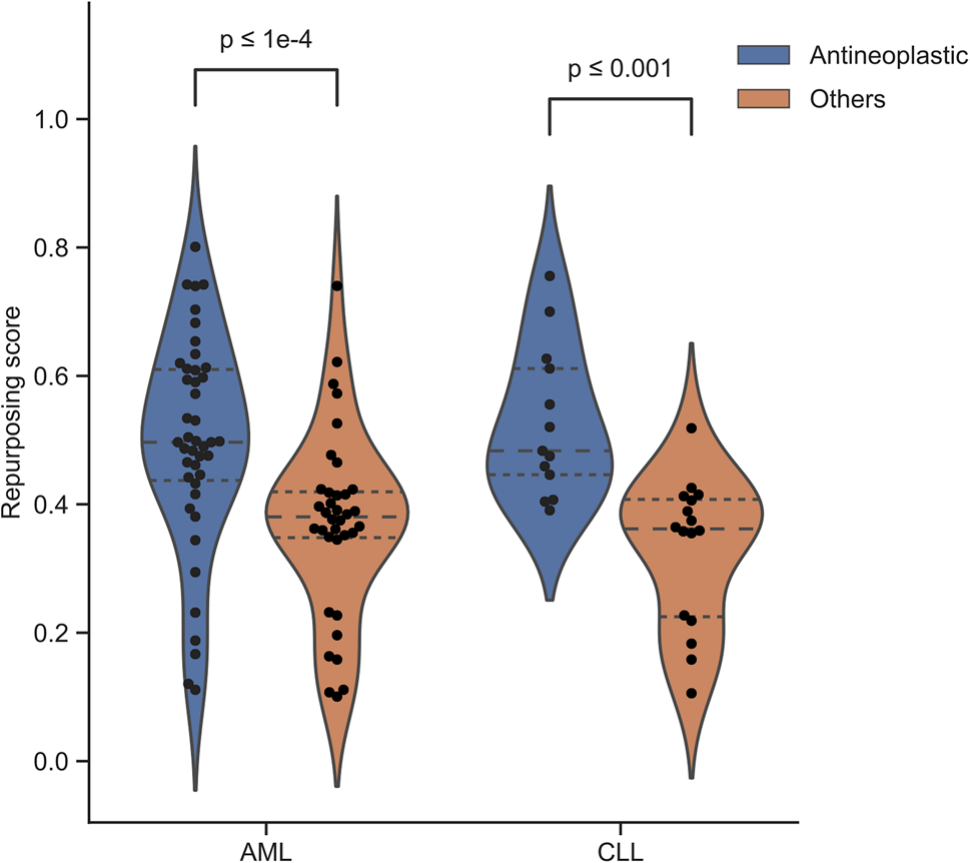
Distribution of DrugRepo scores for drugs with similar therapeutic uses (antineoplastic) vs other drugs.

Furthermore, we have investigated whether DrugRepo scores are significantly different for drugs with dissimilar therapeutic uses. Anatomical Therapeutic Chemical (ATC) is a widely recognized system that categorizes drugs into five levels. ATC level two group drugs/compounds into 94 classes based on therapeutic effects. The ChEMBL database provides ATC level two classifications for 2,739 drugs/compounds. The 'antineoplastic' is one of the classes (out of 94) for ATC level two. Antineoplastic drugs are mostly used for the treatment of cancers. We, therefore, have tried to compare the distributions of Antineoplastic drugs with other drugs across two cancer types, i.e., Acute Myeloid leukemia and Chronic lymphocytic leukemia. The distributions of antineoplastic drugs have been significantly higher than other drugs, with *P* values < 0.001 for both cancer types (Fig. 5). Therefore, we can claim that drugs with similar therapeutic uses have higher scores compared to any random drugs.

### Matching DrugRepo candidate compounds with drugs in clinical trials

To establish if the DrugRepo pipeline enriches compounds already tested in patients, we have chosen nine commonly studied cancer types to match repurposed compounds in DrugRepo with completed Phase I or Phase II or ongoing Phase III clinical trials. First, we have obtained the names of all compounds that match these criteria using the clinical trial API. Using PubChem's API, we have tried mapping the compound names with standard InChIKeys. However, the naming convention for compounds still needs to be standardized, and several compounds have not been mapped and therefore omitted from the subsequent steps.

**Figure 6:**
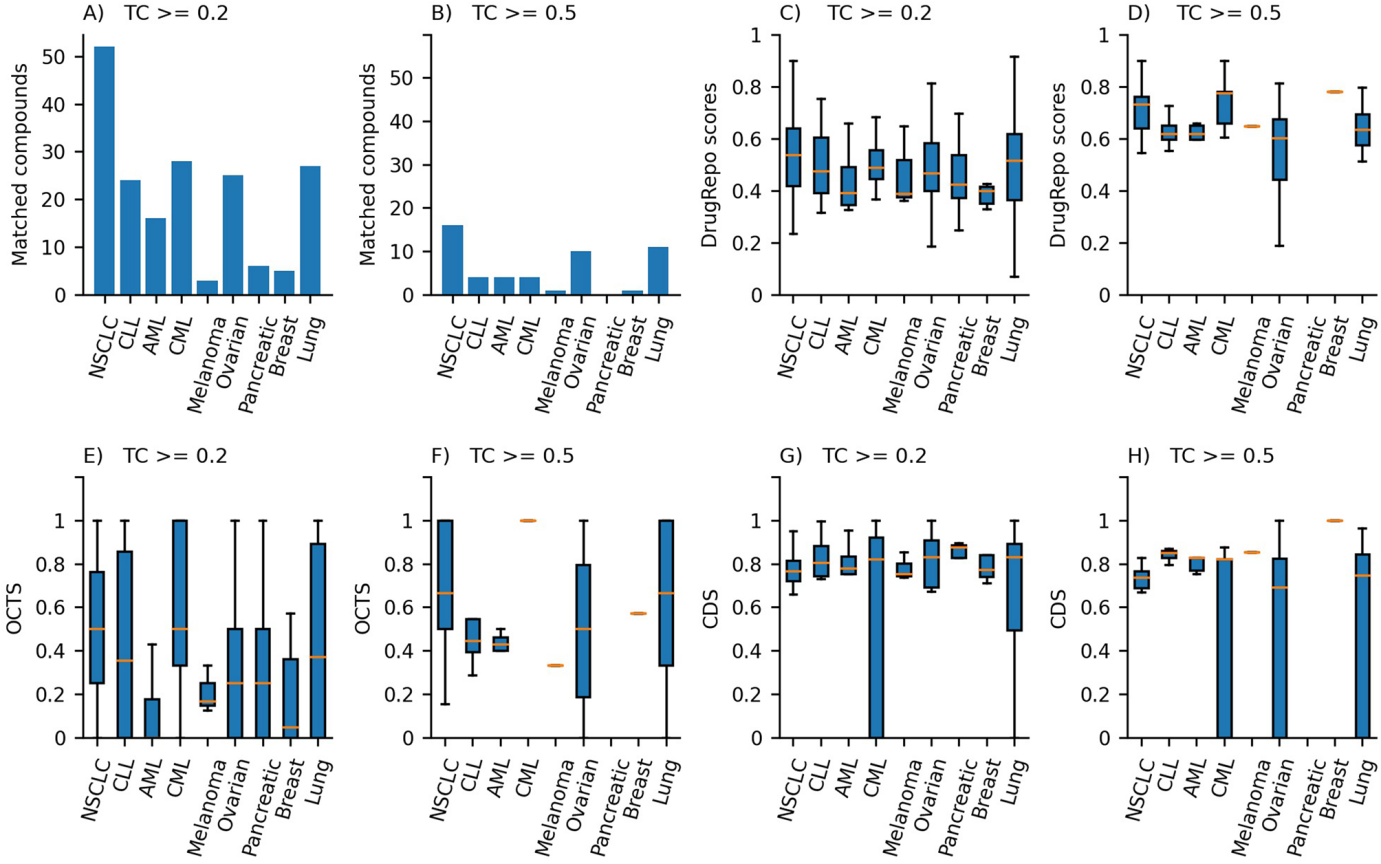
(**A**) The number of matched candidate compounds across nine cancer types using structural similarity (TC) ≥ 0.2, (**B**) Number of matched candidate compounds using structural similarity (TC)  ≥ 0.5, (**C**) Distribution of DrugRepo scores for matched compounds for structural similarity (TC)  ≥  0.2, (**D**) Distribution of DrugRepo scores for matched compounds for structural similarity (TC)  ≥  0.5. (**E**) Distribution of compound-disease score for repurposed compounds TC  ≥  0.2, (**F**) Distribution of compound-disease score for repurposed compounds TC  ≥  0.5, (**G**) Distribution overlapping target profile scores between approved and repurposed compounds TC  ≥  0.2, (**H**) Distribution overlapping target profile scores between approved and repurposed compounds TC  ≥  0.5.

Figure 6A, B shows that 186 and 51 candidate compounds using structural similarity thresholds of TC ≥ 0.2 and TC ≥ 0.5, respectively. The names and identifiers of these matched compounds and other statistics are provided in Supplementary file 5.

All these matched indications have either successfully passed phase I or phase II. Some candidate compounds could have high DrugRepo scores but cannot match with compounds at clinical trials. This could be either due to the lack of mapping for some compounds (at clinical trials) or because only limited compounds are being tested at clinical trials. However, by looking at the matched candidate compounds, we may find a suitable threshold on the DrugRepo score to identify repurposing compounds.

Lowering the structural similarity threshold increased the chances of matching more repurposed compounds. Figures 6A, B show the number of matched compounds across the nine cancer types for TC ≥ 0.2 and TC  ≥ 0.5, respectively. Figures 6C and 6D shows the distribution of DrugRepo scores using TC  ≥ 0.2 and TC  ≥ 0.5. Most matched candidate compounds have a median DrugRepo score higher than 0.4. Hence, we have used DrugRepo  ≥ 0.4 to get maximum repurposing compounds.

Furthermore, we have explored the effect of structural similarity on OCTS and CDS. Figure 6E, F show that structural similarity slightly affects the OCTS thresholds but has no significant impact on compound-disease scores (CDS). However, TC values affect the final DrugRepo score (higher structural similarity corresponds to higher DrugRepo scores), as shown in Fig. 6C, D. Matched compounds for most cancer types have median CDS  ≥ 0.7, showing the importance of CDS in defining DrugRepo (Fig. 6E, F). The median of OCTS for different cancer types is lower (0.1–0.5) than CDS because most of the compound’s complete target profiles (across the entire druggable genome) are not experimentally tested. The average number of targets for the candidate and approved compounds for each of the five databases is less than 7^30^. However, with the availability of additional high throughput DTI studies, the distribution of OCTS in DrugRepo score may also increase.

**Figure 7:**
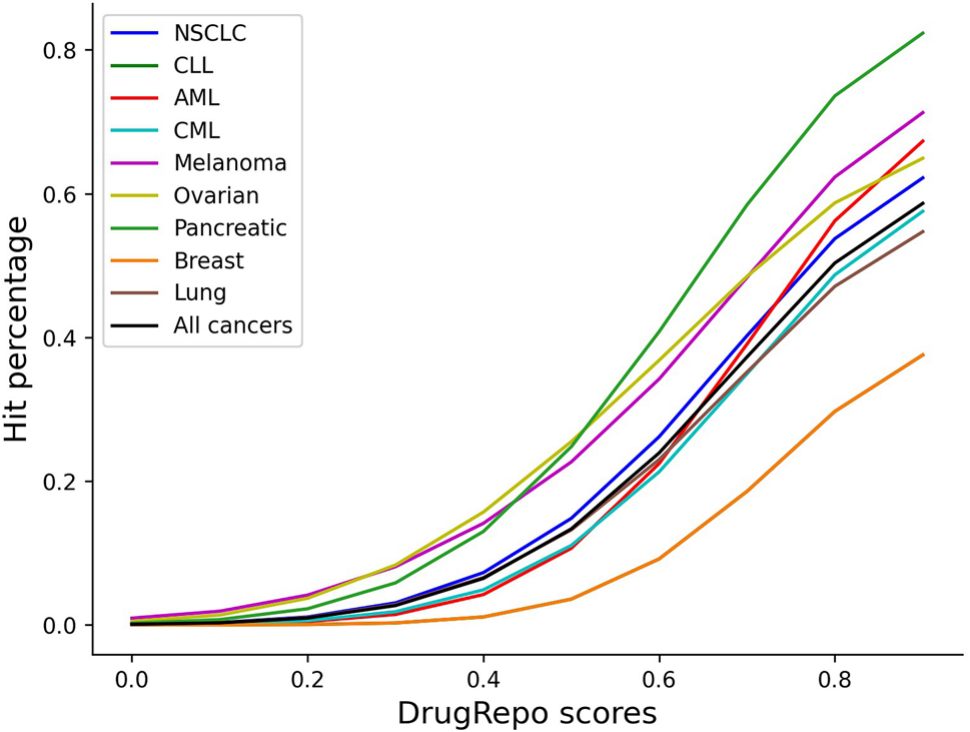
Hit ratio at different thresholds on different DrugRepo scores.The X-axis shows thresholds on DrugRepo scores, and Y-axis shows the percentage of hits while matching with repurposed compounds at https://clinicaltrials.gov/.

Setting a lower threshold on DrugRepo scores may result in more false positives (compounds not in clinical trials). So, we have analyzed the proportion of hits (compounds in clinical trials) across ten thresholds on DrugRepo scores. As shown in Fig. 7, the hit percentage is greater than 5% (for all cancers) at DrugRepo score  ≥ 0.4. However, having miss-hits with clinical trials does not necessarily mean false positives, as clinical trials contain a limited number of studies and compounds.

Based on these successful matching, a DrugRepo score  ≥ 0.4 might guarantee the repurposing of a candidate compound with fewer chances of false positives. Using the threshold of DrugRepo score ≥ 0.4, we have specifically checked how many approved drugs could be repurposed. Surprisingly, we have been able to repurpose 516 drugs across 545 diseases (6229 pairs) from various groups, including cancers, lung, kidney, skin, and eye diseases. Table 1 shows the repurposed drugs having an average DrugRepo score  ≥ 0.9, along with the actual and predicted indications and the ongoing phase of clinical trials. There are nine drugs in Table 1 repurposed for ten indications. The complete table containing 516 drugs repurposed for 545 diseases, along with average DrugRepo scores, is provided in Supplementary file 6. Only a few compounds are tested in clinical trials; therefore, we suggest that top-scoring repurposed drugs test in-vitro to evaluate the significance of DrugRepo scores in future work.

**Table 1:** Drugs repurposed by DrugRepo at average score  ≥  0.9.

Drug	Repurposed disease name	Actual indications	Average DrugRepo score	Clinical trial phase
Clobetasol	Keratitis	Psoriasis	0.95	NA
Everolimus	Renal cell carcinoma	Subependymal giant cell astrocytoma; Breast adenocarcinoma	0.93	Completed phase 3 (NCT01865747, NCT01668784)
Temsirolimus	Subependymal giant cell astrocytoma	Renal cell carcinoma	0.93	Completed phase 3 (NCT01865747)
Temsirolimus	Breast adenocarcinoma	Renal cell carcinoma	0.92	Completed phase 2 (NCT01111825)
Clobetasol	Chronic myeloproliferative disorder	Allergic rhinitis	0.91	NA
Paliperidone	Developmental disabilities	Schizoaffective Disorder	0.91	Completed phase 3 (NCT00549562)
Alisporivir	Dry eye syndromes	Hepatitis C	0.91	Nil
Terlipressin	Shock	Hepatorenal syndrome	0.91	Completed phase 2 (NCT00481572)
Valproic acid	Bipolar I disorder	Seizures	0.9	Completed phase 1 (NCT01094249)
Ethinyl estradiol	Memory disorders	Turner syndrome	0.9	NA
Ethinyl estradiol	Myocardial infarction	Turner syndrome	0.9	NA

### Using the DrugRepo’s GUI to repurpose drugs for CML

**Figure 8:**
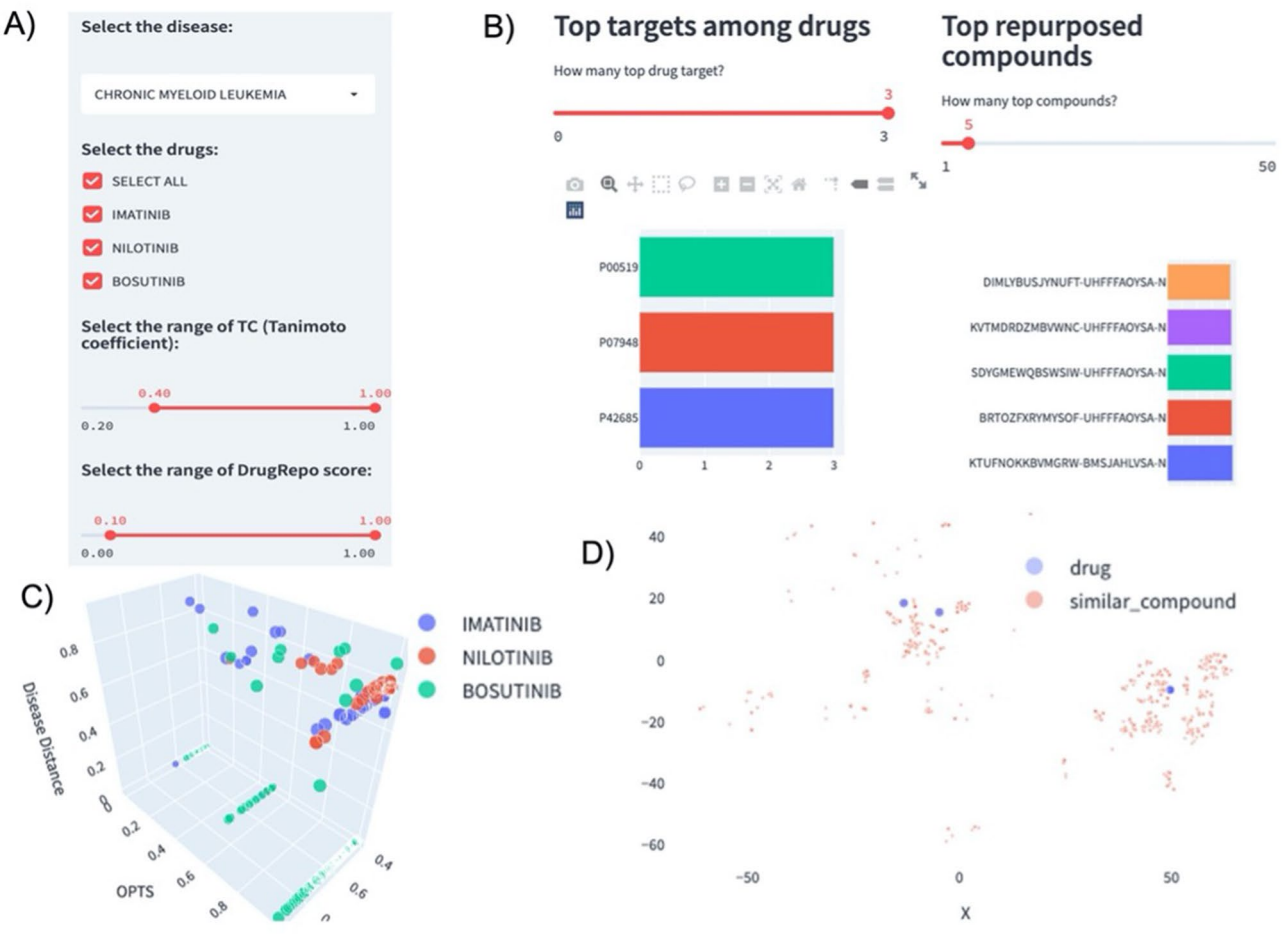
Using the DrugRepo GUI to repurpose drugs for CML. (**A**) Select disease interface, users can uncheck any of the approved drugs, modify TC, and DrugRepo thresholds, (**B**) Top targets for approved drugs and top repurposing/rescuing compounds, (**C**) Drug repurposing plot, each point is based on three scores for an approved drug and repurposing compound, (**D**) The 2D visualization for structural similarities between approved drugs and repurposing compounds.

We provide a case study on Chronic Myeloid Leukaemia (CML) using the web interface at http://drugrepo.org/. DrugRepo has three approved drugs (imatinib, nilotinib, and bosutinib) for CML. Users may check one or more of these approved drugs and customize the structural similarity and DrugRepo thresholds, as shown in Fig. 8A. The figure’s section on DrugRepo displays the results for the selected disease and associated approved compounds. Three figures can be selected from the figures dropdown list, i.e., (1) Statistics, (2) Repurposing scores, and (3) TSNE. The ‘Statistics’ displays the top targets (for approved drugs) and a list of repurposing compounds (Fig. 8B). We have used standard InChIKey identifiers for the repurposing compounds instead of names because many preclinical and investigational compounds aren’t assigned proper names. For targets, we have used UniProt IDs. The ‘Repurposing scores’ provide a 3D scatterplot with the X-axis as TC, the Y-axis as OCTS and the Z-axis as CDS scores. Each point is a pair of approved drug and repurposed compound. Repurposing compounds are assigned the colours like the most associated approved drug (Fig. 8C). More details about diseases, approved drugs, repurposing compounds, and the three scores will show if users click on the scatter points. T-distributed stochastic neighbour embedding (TSNE) is used to visualize the 2D similarity between approved drugs (purple) and repurposing compounds (red), as shown in Fig. 8D. The 2D similarity has been computed based on ECFP4 fingerprints.

Down to the Figures, there is a Tables section containing four tables in the ‘Tables’ dropdown list as listed below:

- Drug repurposing table.
- Approved drugs for the disease.
- Disease-gene associations.
- Drug target profiles for drugs and compounds.

The ‘Drug Repurposing’ table provides four scores (TC, OCTS, CDS and DrugRepo) between approved and repurposed drugs. The ‘Approved drugs for the disease’ table displays UML-CUIs for the selected disease and standard InChIKeys of the approved drugs. The ‘Disease-gene associations’ table displays the genes associated with the selected disease. Finally, the ‘Drug target profiles for drug and compounds’ table provides a list of drug targets associated with the approved drugs and candidate compounds. Users can use filter and sort options to customize the results and download data for further analysis.

## Conclusion

DrugRepo provides a platform for repurposing or rescuing a massive number of compounds using chemical and genomic features. We have evaluated our methods by leveraging the datasets available at clinical trials and CTD. DrugRepo is based on three components, i.e., structural similarity (TC), overlapping compound-target score (OCTS), and compound-disease scores (CDS). CDS (median ≥ 0.75 in nine cancer types) is based on PPI networks and is more effective than OCTS (median ≥ 0.3) for matching repurposed compounds (as shown in Fig. 6G, H). By matching repurposed compounds, we suggest a DrugRepo score ≥ 0.4 as the threshold to repurpose compounds for a selected disease, as it has a lower chance of having false positives (Fig. 7). Based on this threshold, we could repurpose 516 drugs across 545 diseases (6229 pairs) from various groups (Supplementary file 6). As shown in Table 1, drug indications having high DrugRepo scores (> 0.9) are likely to pass trials at high phases of clinical trials. This provides an opportunity for future wet-lab scientists to validate high-scoring repurposed drugs experimentally and may initiate new clinical trials.

Computational analysis is further supported by a web application (http://drugrepo.org/), where users can select a particular disease and get repurposed drugs or rescued compounds along with other parameters. Using DrugRepo GUI, users can select a particular disease, check the approved drugs and targets associated with approved drugs or candidate compounds, and finally download the drugs that can be repurposed for the selected disease. Users can adjust thresholds for structural similarity (TC) and DrugRepo scores (TC), as shown in Fig. 8A. We also provide user-friendly visualization by which users can apply different filters to obtain specific results and finally download results for further analysis. The web application can help design new drug repurposing applications and use existing information for predictive analysis.

However, our method has some limitations. For instance, the proposed approach depends on OCTS between approved drugs and candidate compounds. Missing data in DTI profiles for an approved drug or candidate compound may cause failure to capture some important repurposing candidates. Although we have integrated DTI profiles from the five most comprehensive databases, the average number of targets for approved drugs is around seven, which is much less than expected (as druggable targets are around 1000). However, new releases of DTI databases (such as ChEMBL, DTC, DrugBank or BindingDB) may bring additional curated DTIs, resulting in better repurposing applications. We do updates with the newly curated datasets to make DrugRepo more effective. Presently DrugRepo is based only on three components. However, results can be further improved with more components (such as gene expression data). We will therefore incorporate these improvements in the next version of DrugRepo.

## Supplementary Information

Supplementary Information 1.

Supplementary Information 2.

Supplementary Information 3.

Supplementary Information 4.

Supplementary Information 5.

Supplementary Information 6.

## Data Availability

DrugRepo is available at http://drugrepo.org/.
